# Assessing Macro Disease Index of Wheat Stripe Rust Based on Segformer with Complex Background in the Field

**DOI:** 10.3390/s22155676

**Published:** 2022-07-29

**Authors:** Jie Deng, Xuan Lv, Lujia Yang, Baoqiang Zhao, Congying Zhou, Ziqian Yang, Jiarui Jiang, Ning Ning, Jinyu Zhang, Junzheng Shi, Zhanhong Ma

**Affiliations:** MARA Key Lab of Pest Monitoring and Green Management, Department of Plant Pathology, College of Plant Protection, China Agricultural University, Beijing 100193, China; djcc@cau.edu.cn (J.D.); lvxuan613@outlook.com (X.L.); lujiayang@cau.edu.cn (L.Y.); sy20213193078@cau.edu.cn (B.Z.); zhoucy@cau.edu.cn (C.Z.); solitary@cau.edu.cn (Z.Y.); 2020319010121@cau.edu.cn (J.J.); 2020319010111@cau.edu.cn (N.N.); 2020319010117@cau.edu.cn (J.Z.); 2021319010116@cau.edu.cn (J.S.)

**Keywords:** WSR, disease index, plant diseases, semantic segmentation, class imbalance

## Abstract

Wheat stripe rust (WSR) is a foliar disease that causes destructive damage in the wheat production context. Accurately estimating the severity of WSR in the autumn growing stage can help to objectively monitor the disease incidence level of WSR and predict the nationwide disease incidence in the following year, which have great significance for controlling its nationwide spread and ensuring the safety of grain production. In this study, to address the low accuracy and the efficiency of disease index estimation by traditional methods, WSR-diseased areas are segmented based on Segformer, and the macro disease index (MDI) is automatically calculated for the measurement of canopy-scale disease incidence. The results obtained with different semantic segmentation algorithms, loss functions, and data sets are compared for the segmentation effect, in order to address the severe class imbalance in disease region segmentation. We find that: (1) The results of the various models differed significantly, with Segformer being the best algorithm for WSR segmentation (rust class F1 score = 72.60%), based on the original data set; (2) the imbalanced nature of the data has a significant impact on the identification of the minority class (i.e., the rust class), for which solutions based on loss functions and re-weighting of the minority class are ineffective; (3) data augmentation of the minority class or under-sampling of the original data set to increase the proportion of the rust class greatly improved the F1-score of the model (rust class F1 score = 86.6%), revealing that re-sampling is a simple and effective approach to alleviating the class imbalance problem. Finally, the MDI was used to evaluate the models based on the different data sets, where the model based on the augmented data set presented the best performance (R^2^ = 0.992, RMSE = 0.008). In conclusion, the deep-learning-based semantic segmentation method, and the corresponding optimization measures, applied in this study allow us to achieve pixel-level accurate segmentation of WSR regions on wheat leaves, thus enabling accurate assessment of the degree of WSR disease under complex backgrounds in the field, consequently providing technical support for field surveys and calculation of the disease level.

## 1. Introduction

Wheat stripe rust, caused by *Puccinia striiformis* f. sp. *tritici* (*Pst*), is a fungal disease that causes serious damage to wheat production worldwide [1,2], especially in some temperate regions [3]. The development and spread of WSR can be very rapid under low temperature and high humidity conditions, such as moderate spring rainfall in the 5–24 °C range. It has been estimated that global yield losses due to WSR are at least 5.47 million tons per year [4]. The most severe epidemic years of WSR in China were 1950, 1964, 1990, 2002, and 2017, with the first four instances causing 6 million tons, 3.2 billion million tons, 1.24 million tons, and 0.85 million tons of yield loss, respectively [5]. In 2017, the occurrence area of WSR in China was about 5.56 million hm^2^, similar to the heavy year of 2002, but the final disease index and yield losses were significantly less than those in 2002 [6].

The annual cycle of WSR is completed with the long-distance transmission of uredospores. WSR is not resistant to high temperatures and prefers cooler temperatures; thus, in areas where the average ten-day summer temperature is greater than 23 °C, the rust fungus cannot survive over-summering. Therefore, the survival of summer is a key stage in the annual cycle of WSR. The over-summering region of WSR in China is mainly concentrated in the cool northwest region, which provides a fungal source for the winter production region with relatively high winter temperatures. In winter, *Pst* continues to infect and spread in winter production areas, increasing the fungal population, and becoming the source to infect the northern wheat region in the following year, thus playing a key role in the prevalence of WSR in spring [7]. Pu et al. [8] have found that there exists a very significant positive correlation between the incidence area of autumn seedlings in Gansu province and the nationwide area of occurrence in the following year. Therefore, every winter in China, a large number of plant protection workers visit the associated production area to investigate the WSR disease index, in order to predict the disease index of the following year and to provide reference for the development of prevention and control measures in advance. In the traditional field investigation of WSR, when many points need to be investigated, the average disease index is usually estimated by direct visual observation (GB/T15795-2011). However, due to the different standards of artificial evaluation, which are highly subjective, the results of such surveys are greatly differ, resulting in low representativeness of the collected data. Therefore, it is particularly important to find an objective and automatic method to identify the disease index of WSR in autumn wheat. This not only can standardize the field investigation process, but will also greatly improves statistical efficiency and reduce the investigation workload.

Compared with traditional machine algorithms, deep-learning algorithms have shown superior and advanced performance in different fields in recent years, and have achieve better results when applied for plant disease identification [9,10]. For example, Lin et al. [11] have identified wheat leaf diseases, including powdery mildew, bacterial streak, leaf blight, leaf rust, and stripe rust, based on the use of a convolutional neural network model, and achieved an accuracy of 90.1%. However, considering current deep learning applications, few studies have focused on developing quantitative disease stress severity assessment systems. Liang et al. [12] have developed a multi-functional diagnostic system (PDSE-S-2-NET) to achieve plant species identification, disease classification, and severity estimation, which achieved an overall accuracy of 91% for disease severity estimation. Esgario et al. [13] have developed a coffee leaf disease severity estimation model based on a convolutional neural network, which obtained an accuracy of 86.51%. Hayit [14] has used a deep-learning method to determine the severity of WSR, presenting an accuracy of 91%. The above studies proposed new ideas and methods for disease severity calculation, but these were limited to a single plant leaf, and the images used in the experiments were based on a single background image under laboratory conditions; therefore, the practical application scope of disease investigation under complex backgrounds such as those common in the field remains limited. Mi et al. [15] have used images of wheat leaves with complex field background and applied a deep-learning algorithm to assess the severity of WSR with an accuracy of 97.99%, which has significance in the identification of wheat disease resistance, but their approach was still limited to the single leaf scale. The automatic identification of WSR at the tillering stage based on RGB images containing a large number of leaves (e.g., tens to hundreds) at canopy scale has not previously been reported.

One of the key problems posed by images involving a large number of leaves is severe class imbalance; that is, the rust class occurs in a very low percentage of leaves. An imbalanced data distribution often occurs in image segmentation tasks, such as when conducting defect detection of industrial products, road extraction, and lesion region extraction. Unbalanced data poses a great challenge in classification model construction [16]. Most classification models are built on the premise of balanced data sets, and minority samples may be considered as noise by the learning model [17]. The minority examples usually overlap with other regions where the prior probabilities of the two classes are almost equal [18]. Therefore, in this study, we explore solutions to the imbalanced data problem, in order to improve model performance and provide ideas for similar situations. We collected images of WSR-diseased leaves at the canopy scale in the autumn tillering stage under a complex background in the field, then implemented a deep convolutional neural network model to semantically segment the WSR in the images and automatically calculate the macroscopic disease index, in order to improve the measurement efficiency and accuracy. First, we manually annotated and segmented (in a non-overlapping manner) all of the images, obtaining a total of 25,530 images of size 256 × 256 pixels to construct the original data set. Second, to address the severe class imbalance problem, we performed data augmentation for the minority class, as well as down-sampling on the original data set. In this way, we constructed an augmented dataset and three under-sampled data sets to improve the class balancing, and then compared the modelling effects with the different data sets. Finally, we compared the performance of different deep learning semantic segmentation model algorithms and different loss functions, in order to choose the best combination for model construction.

## 2. Materials and Methods

**Figure 1:**
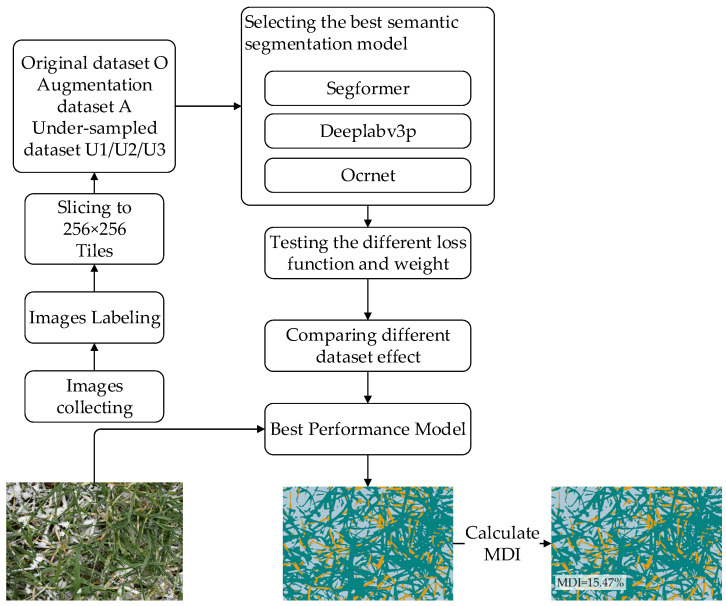
Flowchart of data analysis and processing.

A flowchart detailing the data analysis and processing is shown in Figure 1. The image acquisition process is described in Section 2.1 of this paper, while the methods and processes are described in Section 2.2, Section 2.3, Section 2.4, Section 2.5, Section 2.6.

### 2.1. Data Sources

**Figure 2:**
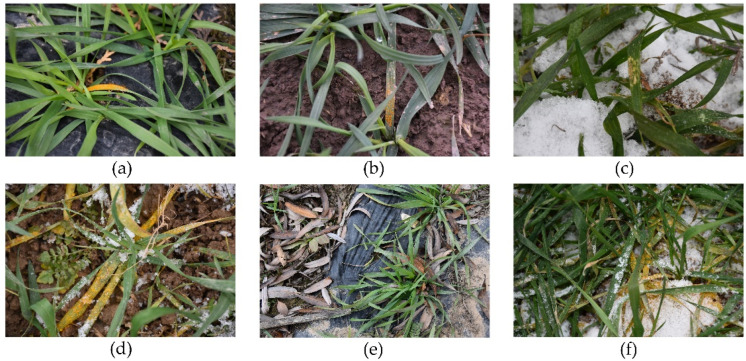
Examples of winter wheat infected by stripe rust with complex backgrounds: (**a**) film mulching; (**b**) soil; (**c**) snow cover; (**d**) weeds; (**e**) dried leaves; and (**f**) overlapping.

The images were collected with a Nikon camera (NIKON D5600, Nikon Corporation, Tokyo, Japan.) in late November 2020 from infected fields in Gangu County, Tianshui City, Gansu Province, China (between 104°58′ and 105°31′ E longitude and 34°31′ and 35°03′ N latitude). At this time, the average daily temperature in the area was between −2 °C and 3.5 °C, and winter wheat started to move from the tillering stage to the over-wintering stage. To ensure the heterogeneity of the images, image acquisition was taken vertically at 0.3–1 m above the wheat canopy. Images were collected under a complex field background (e.g., mulch films, snow, soil clods, fallen leaves, weeds, overlapping leaves), as shown in Figure 2. The complex background makes the data set more heterogeneous, such that the constructed model can be applied to different field situations, but this also greatly increases the difficulty of leaf segmentation.

**Figure 3:**
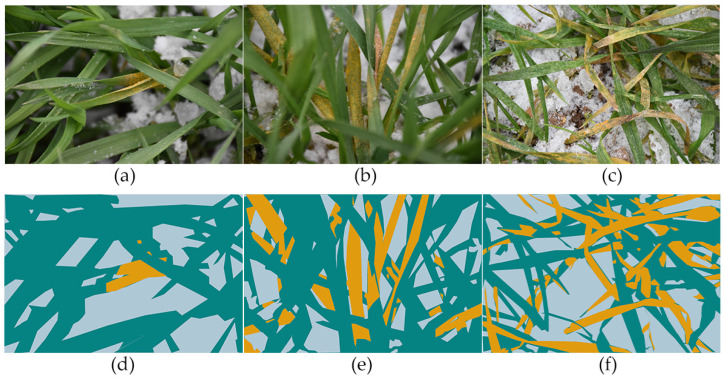
(**a**–**c**) Three samples of wheat leaves; and (**d**–**f**) annotation of healthy wheat (blue) and infected wheat (yellow) areas.

To train a convolutional neural network to identify disease areas on leaves, the images first need to be annotated. In this study, all WSR images were manually annotated using the Labelme [19] software. Figure 3a–c show some of the WSR images in this study, and the corresponding Figure 3d–f show the annotated images for each sample. The pixels in the diseased areas of wheat leaves are annotated in yellow and marked as the rust class, while the remaining healthy areas of wheat leaves are annotated in dark blue and are labelled as the healthy class. Other background areas, such as soil, snow, mulch films, and dead leaves, are annotated in light blue and marked as the other class.

### 2.2. Data Preprocessing

A total of 370 images of size 3000 × 2000 pixels and corresponding labels were used in this study, of which 70 pairs were randomly selected for independent visual inspection and MDI calculation data. Due to the large image size, it was inconceivable to use them directly as an input to the deep-learning model. Thus, the remaining 300 pairs were segmented using a size of 256 × 256 pixels without overlapping. A total of 25,530 pairs of images and corresponding labels were obtained to form the original data set, which was randomly divided into training, validation, and test sets at a ratio of 0.6:0.2:0.2.

**Figure 4:**
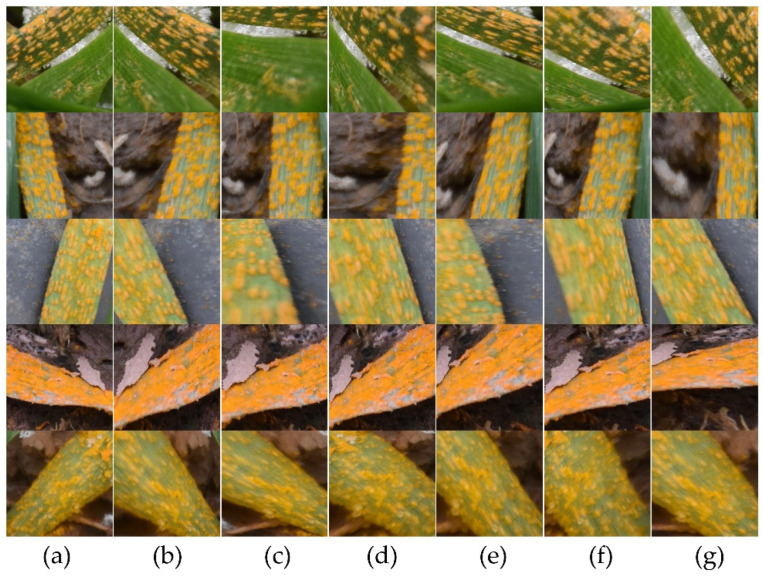
Example images of data augmentation, (**a**) the original image, (**b**–**g**), the augmentation data.

The segmentation of disease regions is essentially a multi-classification problem for each pixel in the image. However, if the total number of pixels in diseased regions is much smaller than in non-diseased regions, this creates a class imbalance problem, which leads to the lower accuracy of the neural network on the minority class(es) [20]. We considered each labelled image to determine statistics, and found that the proportion of the rust class in the whole data set was very low (5.66%), far lower than the healthy or other classes (>46.30%), making it a typical class-imbalanced data set. In order to avoid the data leakage problem [21] in the data augmentation process, we split the data sets before the data augmentation step. Therefore, we first divided the original dataset O into training, validation, and test sets in the ratio 0.6:0.2:0.2. Then, we performed data augmentation on the training and validation sets, respectively. We filtered out the tiles with rust area > 30% and used the Augmentor image enhancement library to enhance the data, with the main operations being random rotation (−10° to 10°), random zoom (0.85–1.15), perspective transforms, and elastic distortions (Figure 4). A total of 18,000 paired samples were generated. These samples were added to the original dataset, for a total of 43,530 pairs, and the percentage of the rust class increased to 17.89% in the augmented dataset A. We constructed the under-sampled data sets U1, U2, and U3 by randomly sampling different proportions of images containing only the healthy class, only the other class, and both of the healthy and other classes on the basis of the original data set. The details of the percentage of each class in the various data sets are shown in Table 1.

### 2.3. Semantic Segmentation Model

We used the Segformer [22] semantic segmentation framework for model construction. Segformer uses Transformers as encoders and lightweight multi-layer perceptrons (MLPs) as decoders, which makes it simple and efficient, yet powerful, as it does not require positional encoding and complex decoders. Segformer has presented state-of-the-art efficiency, accuracy, and robustness on three publicly available data sets: Cityscapes, ADE20K, and COCOStuff [22]. We also compared its performance with that of other advanced semantic segmentation algorithms, including OCRNet [23], Deeplabv3+ [24], PSPNet [25], DNLNet [26], FCN [27], GCNet [28] and SFNet [29]. Among them, Segformer is a Transformer model, while the rest are convolutional neural network (CNN) models. 

To solve the problem of a class-imbalance in the data set, we compared the performances of various loss functions, including cross-entropy loss (CE), dice loss, boundary loss [30], ohem-cross-entropy loss [31], Lovász-Softmax loss [32], and focal loss [33], which are considered to perform better in cases characterized by difficult sample identification and class imbalance problems.

### 2.4. Model Training

Experiment 1: Comparison of different semantic segmentation algorithm frameworks. We compared the recognition effects of different semantic segmentation algorithms based on the original data set, where the modelling parameters were as shown in Table 2. 

Experiment 2: Comparison of different loss functions. We used the same parameters and different loss functions, based on the best algorithmic framework of Experiment 1 and the original data set, in order to test the impact of the loss function in modelling an imbalanced data set. We also compared the impacts of different weights of the rust class in cross-entropy loss.

Experiment 3: Comparison of different data sets. We built the model using the best algorithmic framework and loss function based on the five data sets constructed by under-sampling and data augmentation, in order to compare the impacts of the different data sets.

All data sets were divided into training, validation, and test sets in the ratio of 0.6:0.2:0.2. Each epoch was tested using the validation set, in order to preserve the best model. The model performance was ultimately measured based on the test set. During training, a real-time data augmentation technique was applied, where each batch of images was used to generate new images, which were then input into the CNN network. Real-time data amplification was carried out using resize-step-scaling (0.75–1.25), random horizontal flip, and random distort (brightness-range: 0.4, contrast-range: 0.4, saturation-range: 0.4). During network training, the model with the lowest loss calculated on the validation set data was saved. All experiments were performed on an Nvidia GeForce GTX 3090 GPU, using the PaddleSeg 2.3 [34] image segmentation development kit in CUDA 10.2 and the Paddle framework (Baidu deep learning open source library).

### 2.5. Model Evaluation

The precision, recall, F1 score, and intersection over union (IoU) metrics were used to evaluate the performance of the preserved models on the test data set, which were calculated as follows.

(1) $$\mathrm{Precision}=\frac{TP}{TP+FP\;}$$

(2) $$\mathrm{Recall}=\frac{TP}{TP+FN\;}$$

(3) $$F_{1}-\mathrm{score}\;=2\times \frac{\mathrm{Precision}\times \mathrm{Recall}}{\mathrm{Precision}+\mathrm{Recall}}$$

(4) $$\mathrm{IoU}=\frac{\mathrm{Area}\;\mathrm{of}\;\mathrm{Overlap}\;}{\mathrm{Area}\;\mathrm{of}\;\mathrm{Union}\;}$$

where *TP* denotes the true positives, *FP* denotes false positives, and *FN* denotes false negatives. Based on these three metrics, we applied independent test set data to evaluate the performances with different models, different loss functions, and different data sets.

### 2.6. Calculation Method of Macro Disease Index (MDI)

**Figure 5:**
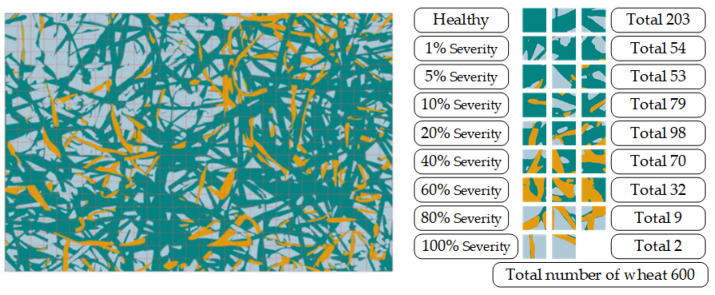
MDI calculation process example diagram (Yellow represents infested wheat, blue represents healthy wheat): first, the image is divided into small units of a certain size. Then, statistics are calculated based on these tiles.

After the identification and segmentation healthy and rust class regions, it was still impossible to quantify the occurrence of the disease (e.g., incidence, severity level of disease, disease index, and so on), in order to predict the prevalence of plant disease and, subsequently, evaluate the yield loss. According to the “Rules for monitoring and forecast of the WSR” (GB/T 15795-2011), the disease index is a comprehensive index of the incidence and severity level of the disease that is used to indicate the average level of the disease. The incidence is the percentage of diseased leaves in the total number of investigated leaves, which is used to indicate the prevalence of disease. The severity level is the average percentage of the area of lesions on the diseased leaves in the total area of leaves. It is found that traditional disease index investigation methods are based on a single leaf, and the severity of a single leaf can be accurately calculated from images containing only a single leaf [14,15]. At the canopy scale, as the wheat leaves tend to overlap each other, it is difficult to identify and count the number of leaves. Therefore, we used the macro disease index (MDI) to represent the average level of disease occurrence in the specified area, which was calculated according to formula (5):

(5) $$D=\frac{\sum^{\;}\left(i\times L_{i}\right)}{nL}\;$$

where D is the macro disease index; i is the macro severity level of disease, which is the proportion of the WSR area to the wheat area per unit, with 8 grades (1%, 5%, 10%, 20%, 40%, 60%, 80%, and 100%); $L_{i}$ is the number of units at macro severity level grade *i*; *n* is the highest-level representative value (here, it is 100%); and L is the total number of units containing wheat classes. For example, as seen in Figure 5, the resolution of the original image in this study is 3000 × 2000 pixels, which means that the actual area of the ground was about 1.2 square meters. This is divided into 600 unit areas (each of size 100 × 100 pixels), and the unit areas containing wheat classes (healthy class + rust class) were counted one by one, as well as the macro severity level value per unit (area), and its corresponding quantity, in order to calculate the macro disease index, which is used to represent the average level of disease occurrence in a certain area.

The macro disease index represents the overall level of disease occurrence per unit area. In the actual field investigation process, the macro disease index can be automatically calculated by standardizing pictures (e.g., with fixed height, ground area, and shooting angle) and identifying the pictures during the actual field survey. In this study, the macro disease index was used to measure the model prediction results. The cold of the MDI calculation will be available at https://github.com/caudjcc/Micro-DI (accessed on 2 July 2022).

## 3. Results and Analysis

### 3.1. Results with Different Models 

**Figure 6:**
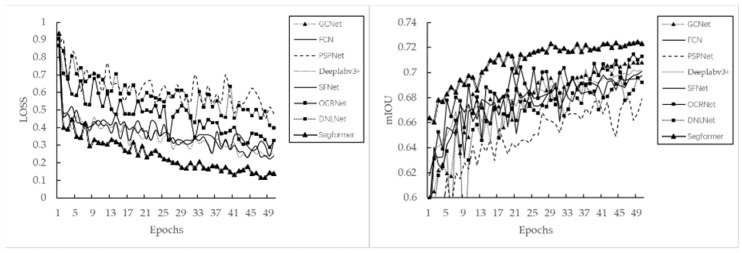
The training loss and mIoU of the different frameworks during model training (one validation per epoch).

The original data set and cross-entropy loss function were used to construct the baseline model, in order to assess the performance of the different algorithmic models. The results of the training process are shown in Figure 6. Segformer presented the smallest and fastest decreasing training loss and the highest mIoU in each epoch of the validation process.

The final testing results on the test data sets are detailed in Table 3. There is no significant difference in recognition of the healthy class between the different models (F1-score minimum 87.2%, maximum 89.3%). However, recognition of the rust class significantly differed, with the F1-score being only 64.8% at the lowest and 72.6% at the highest. The results indicate that the framework that achieved the best recognition results for the rust class is Segformer, with both precision and recall ranking highly.

### 3.2. The Influence of Different Loss Functions and Minority Class Weighting 

**Figure 7:**
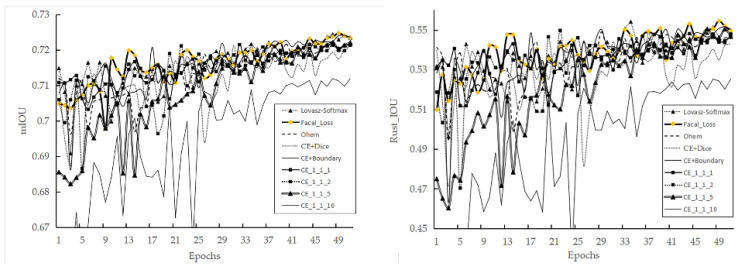
The mIoU and rust class IoU for the validation set obtained when using different loss functions during model training (one validation per epoch).

The average proportions of the rust, healthy, and other classes in all images in this study being 5.66%, 48.03%, and 46.30%, respectively. Such serious class imbalance causes the used model to be much less effective in recognizing the minority class. We chose the best-performing Segformer_ViT_B5 algorithm from the previous step to continue modelling with different loss functions. We used the model parameters determined in the previous step of the experiment as pre-training parameters. For 50 epochs of training, we only changed the loss function, with other parameters remaining unchanged. In addition to testing different loss functions, we also tested the class weights of the CE loss function specifically, modifying the class weights such that the other:healthy:rust ratio changes from 1:1:1 to 1:1:10. As the boundary and dice loss functions had difficulty in converging, we used mixed loss functions, where the ratio of CE to the other loss functions is 1:1. The results in Table 4 show that, without any changes to the deep-learning algorithm framework and data set (i.e., using the original dataset O), only changing the loss function can improve the F1 score of the difficult classification class (rust class) by 1.2%. The loss functions (Ohem-cross-entropyloss, Lovász-Softmax loss, and focal loss) typically considered for difficult class mining all performed better. Weighting the rust class has some effect, but the model performance dropped significantly when the rust weight are too large (see Figure 7). In general, changing the loss function or weighting of the minority class does not significantly improve the performance of the model.

### 3.3. Model Results with Different Data Sets 

**Figure 8:**
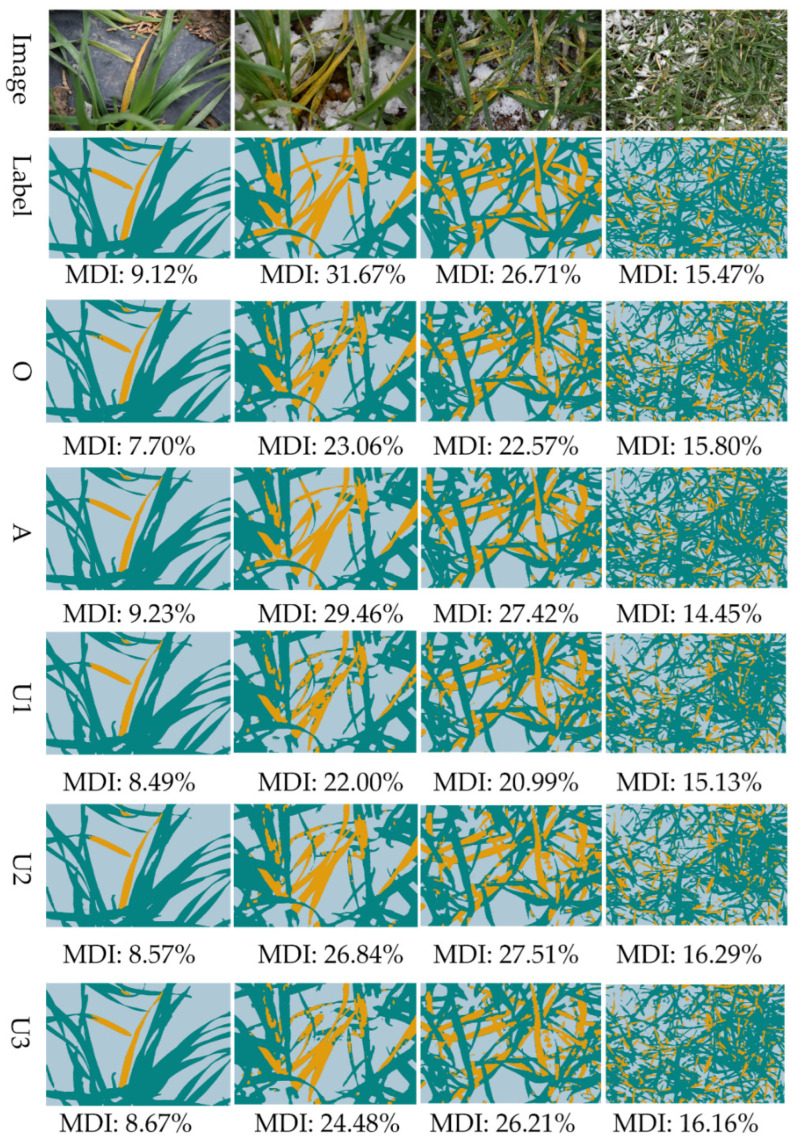
Modelling results for different data sets. O, original data set; A, augmented Data sets; U1, U2, and U3, under-sampled data sets with different proportions. For details of the data sets, see Table 1.

Based on different data sets, the Segformer_ViT_B5 framework and focal loss were adopted for modelling. The results (Table 5) show that the performance of the model was improved substantially as the proportion of the rust class increased. It is found that whether the class is balanced or not has a significant impact on rust class recognition, and the F1 score of the minority class can be significantly improved by increasing the class equilibrium degree, through under-sampling or augmentation. After visual evaluation of the images predicted by the relevant models (see Figure 8), we find that basically all the models achieved better recognition, with the best performance obtained by the augmented data set model, which presented better recognition on leaves with different depths of field (third column). The models built based on the under-sampled data sets presented a small number of false positives in the soil region.

### 3.4. Comparison of Macro Disease Index Results 

**Figure 9:**
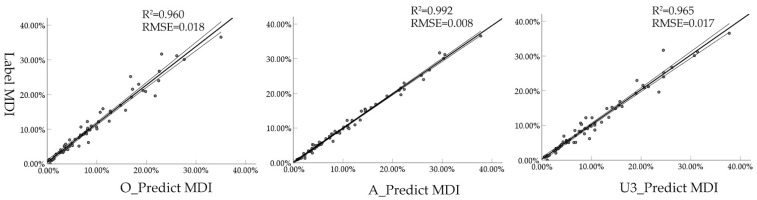
Comparison of MDI based on modeling results of different datasets with reference data MDI.

Seventy original images from the independent test data set were selected and identified using the models built with the different data sets (based on the mixed loss function). Then, the macro disease index of the predicted images was calculated and compared with the results obtained through manual labelling. The results (Figure 9) indicated that the model based on the data set A had the best performance, with a coefficient of determination (R^2^) of 0.992; this outcome is consistent with the visual assessment and F1 score results. This also suggests that the MDI index is suitable as a quantitative indicator describing a range of disease indices based on segmentation results. In practical applications, a certain ground area should be maintained in different pictures.

## 4. Discussion

In this study, we aimed to solve the problem of automatic estimation of the WSR disease index at canopy scale under a complex field background conditions. To solve this problem, the Segformer network structure was applied to construct a semantic model which can achieve the pixel-level semantic segmentation task in complex scenes. Our independent test results demonstrated that the proposed method greatly improves the accuracy of WSR image segmentation, compared with other state-of-the-art models. Compared with the traditional manual investigation method for estimating the plant disease index, we applied the macro disease index and established an automatic recognition method that effectively reduce the error associated with artificial evaluation. In addition, regarding the problem of class imbalance caused by the low infected area proportion in the collected images, the focal loss function was applied to improve the model recognition accuracy. At the same time, the feasibility of improving the rust class proportion through the use of augmented and under-sampled data sets to solve the class imbalance problem was also assessed. This approach allowed the model to achieve outstanding performance. Overall, the developed model achieved the segmentation of WSR-infected areas at pixel level, thus allowing for effective evaluation of disease incidence. As such, it can provide effective technical support for the investigation of WSR in the tillering stage in autumn, which is of great significance for the prediction and forecasting of WSR in China.

Compared to the studies of Hayit and Mi et al. [14,15], in this study, the severity identification approach was not based on a single leaf but, instead, simulated the field investigation process. The data set was captured and constructed based on the state of multiple leaves in the field (from a dozen to hundreds of leaves). Although this increases the difficulty of automatic identification, it reflects the field situation more realistically, and is closer to actual production practice. Patil et al. [35] have adopted a brightness threshold method to segment the spots on leaves, in order to evaluate the severity of several sugarcane fungal diseases, and used the spot to leaf area ratio as an indicator of disease severity level. Lei et al. [36] have used an algorithm to segment stripe rust spots based on spectral images, and obtained the degree of disease index for classification purposes according to the proportion of the area of rust spots in the total leaf area. In this study, the macro disease index was used to achieve quantitative assessment of the disease index at the canopy scale. The definition and realization of the macro disease index not only can facilitate quantification based on canopy data captured using hand-held RGB cameras, but also allows for convenient evaluation of the disease occurrence degree per unit area at the scale of UAV or satellite remote sensing.

The typical feature in this study was the imbalanced class distribution of the original data set, in which the rust class was much smaller than the healthy and other classes. As rare classes occur infrequently, the classification models may have difficulty in predicting minority classes; thus, test samples belonging to such classes are more likely to be misclassified than those belonging to universal classes. In disease detection, the correct classification of samples in minority classes is typically of utmost importance. As has been shown in [37], solving the imbalanced class distribution problem is crucial in visual recognition tasks. When considering minority class image classification, the most typical methods to solve the data imbalance problem are re-weighting/re-sampling methods [16,17,20,38,39,40,41]. In our tests, increasing the weighting of the rust class and the use of different loss functions on our data set does not have a significant improvement effect of the model performance. In contrast, re-sampling is found to a simple and feasible way to greatly improve the model performance. Over-sampling usually severely over-fits rare classes, and under-sampling inevitably leads to reduced CNN generalization ability, due to discarding most of the high-frequency class data. Therefore, we applied data augmentation to the minority class, which increased both the class share and the generalization ability of the model. The outstanding results obtained in our experiments revealed that the use of re-sampling simply and effectively alleviated the class imbalance problem. Recent studies show that using a generative adversarial network (GAN) to generate minority samples can help to solve the data imbalance problem under given conditions [42,43]. In conclusion, data pre-processing techniques, such as data enhancement and generation, for minority classes provide effective solutions for resolving the data imbalance problem.

This study has strong applicability in the agricultural field. Our ultimate goal is to develop a mobile application, with which users can obtain images from the field using portable devices in production, to extract leaf image information, and calculate the macro disease index. The obtained results will be automatically uploaded to a database to provide basic data for the prediction of WSR occurrence in China. However, it is worth noting that this study still had some limitations. First, the images in the data set were taken based on wheat at the autumn tillering stage, mainly for autumn seedling disease investigation. Wheat in the autumn tillering stage has relatively flat leaves, allowing for better estimation of the disease spot area proportion. If the disease degree of wheat is to be investigated after the jointing stage, this model may not be applicable. In addition, the data set used in this study does not contain data on other wheat diseases and insect pests, and other similar disease symptoms (e.g., wheat leaf rust), which may affect the performance of the model. Therefore, leaf damage caused by other types of diseases should be avoided when investigating and taking pictures.

## Figures and Tables

**Table 1:** Details of the percentage of each class in the different data sets.

Percentage of Class	Original Dataset O	Augmentation Dataset A	Under-Sampled Dataset U1	Under-Sampled Dataset U2	Under-Sampled Dataset U3
Healthy	48.03%	42.80%	44.95%	42.66%	38.60%
Rust	5.66%	17.89%	9.37%	12.10%	17.16%
Other	46.30%	39.31%	45.68%	45.24%	44.24%
Total image number	25,530	43,530	15,292	11,792	8292

**Table 2:** Network parameters of the considered architectures.

Optimizer	Momentum	Learning Rate (LR)	LR Scheduler
Sgd	0.9	0.01	Polynomial decay
end_lr	LR_power	Loss function	Iters
0	0.9	Cross-entropy loss	26,000
Mini-batch size	Max epoch	Validation frequency	
30	50	Each epoch	

**Table 3:** Results of different algorithm models.

Models_BACKBONES	Precision			Recall			F1 Score			IOU		
	Other	Healthy	Rust	Other	Healthy	Rust	Other	Healthy	Rust	Other	Healthy	Rust
OCRNet_Hrnetw48	90.3%	86.7%	75.1%	88.2%	90.8%	58.8%	89.2%	88.7%	65.9%	80.6%	79.7%	49.2%
Pspnet_Resnet101	88.1%	86.4%	68.1%	87.3%	88.1%	61.9%	87.7%	87.2%	64.8%	78.0%	77.4%	48.0%
Deeplabv3p_Resnet50	91.1%	87.1%	68.9%	88.1%	90.4%	65.9%	89.6%	88.7%	67.4%	81.2%	79.7%	50.8%
Sfnet_Resnet50	90.4%	87.5%	72.0%	88.8%	89.8%	66.7%	89.6%	88.6%	69.2%	81.2%	79.6%	53.1%
DNLNET_Resnet101	89.1%	87.1%	66.7%	87.6%	88.9%	64.5%	88.4%	88.0%	65.6%	79.2%	78.5%	48.8%
FCN_Hrnetw18	89.7%	86.6%	73.2%	88.2%	89.6%	61.8%	89.0%	88.1%	67.0%	80.1%	78.7%	50.4%
GCNET_Resnet150	88.6%	89.0%	72.4%	90.1%	88.4%	66.5%	89.3%	88.7%	69.3%	80.7%	79.8%	53.0%
Segformer_ViT_B5	91.3%	87.8%	75.6%	88.9%	90.9%	69.8%	90.1%	89.3%	72.6%	82.0%	80.7%	57.0%

**Table 4:** Modeling results of different loss functions.

	Precision			Recall			F1			IOU		
	Other	Healthy	Rust	Other	Healthy	Rust	Other	Healthy	Rust	Other	Healthy	Rust
Ohem	90.9%	87.6%	78.6%	88.9%	90.9%	67.8%	89.9%	89.2%	72.8%	81.6%	80.6%	57.2%
Lovász-Softmax	91.3%	87.7%	77.1%	88.9%	91.1%	68.9%	90.0%	89.3%	72.8%	81.9%	80.7%	57.2%
Focal loss	91.4%	87.5%	77.2%	88.6%	91.3%	69.3%	90.0%	89.4%	73.0%	81.8%	80.8%	57.5%
CE + boundary	91.1%	87.2%	76.2%	88.3%	91.1%	66.5%	89.6%	89.1%	71.0%	81.2%	80.4%	55.1%
CE + dice	90.3%	87.7%	77.7%	89.1%	90.6%	64.9%	89.7%	89.1%	70.7%	81.3%	80.3%	54.7%
CE_1:1:1	92.1%	86.6%	77.6%	87.7%	92.1%	66.9%	89.8%	89.3%	71.8%	81.5%	80.6%	56.1%
CE_1:1:2	91.2%	87.9%	73.8%	88.8%	90.6%	71.4%	89.9%	89.2%	72.6%	81.7%	80.6%	57.0%
CE_1:1:5	91.5%	88.2%	69.2%	88.4%	90.2%	74.3%	89.9%	89.2%	71.6%	81.7%	80.5%	55.8%
CE_1:1:10	91.5%	87.8%	63.4%	87.5%	89.7%	74.5%	89.5%	88.7%	68.5%	80.9%	79.8%	52.1%

**Table 5:** Modelling performance when using data sets with different class equilibrium degree.

DATASETS	Precision			Recall			F1			IOU		
	Other	Healthy	Rust	Other	Healthy	Rust	Other	Healthy	Rust	Other	Healthy	Rust
O	91.4%	87.5%	77.2%	88.6%	91.3%	69.3%	90.0%	89.4%	73.0%	81.8%	80.8%	57.5%
A	90.9%	88.6%	86.5%	90.5%	88.9%	86.7%	90.7%	88.7%	86.6%	83.0%	79.8%	76.3%
U1	92.8%	90.7%	81.5%	92.3%	90.5%	84.0%	92.6%	90.6%	82.7%	86.2%	82.8%	70.6%
U2	93.7%	90.0%	87.2%	92.1%	92.9%	80.9%	92.9%	91.4%	84.0%	86.8%	84.2%	72.4%
U3	93.7%	87.1%	85.1%	90.9%	90.4%	84.1%	92.3%	88.7%	84.6%	85.7%	79.7%	73.3%

## Data Availability

Not applicable.
